# A HPLC fingerprint study on Chaenomelis Fructus

**DOI:** 10.1186/s13065-019-0527-5

**Published:** 2019-01-30

**Authors:** Lili Zhu, Lexia Fang, Zongjin Li, Xiaomei Xie, Ling Zhang

**Affiliations:** 1 College of Pharmacy, Anhui University of Chinese Medicine, Hefei, 230012 China; 2 Institute of Pharmaceutical Analysis, Anhui Academy of Chinese Medicine, Hefei, 230012 Anhui China

**Keywords:** HPLC, Chromatographic fingerprint, Similarity

## Abstract

**Background:**

Chaenomelis Fructus is a type of traditional medicine used in China. At present, the quality standard of Chaenomelis Fructus is mainly based on the content of each component as a control index, lacking overall control. To improve the rapid identification of chemical ingredients for Chaenomelis Fructus, a new approach to the construction for Chaenomelis Fructus is presented in this paper.

**Methods:**

The precision, repeatability and stability of the proposed HPLC method were validated in the study. Twenty batches of Chaenomelis Fructus samples from their geographical origin were analyzed by the HPLC method. Common peaks in the chromatograms were adopted to calculate their relative retention time and relative peak area. The chromatographic data were processed by the Similarity Evaluation System for Chromatographic Fingerprint of Traditional Chinese Medicine software (Version 2004 A) for similarity analysis.

**Results:**

The HPLC method demonstrated satisfactory precision, repeatability and stability. The similarities of the 20 Chaenomelis Fructus samples were 0.967, 0.979, 0.965, 0.992, 0.994, 0.988, 0.974, 0.909, 0.993, 0.894, 0.983, 0.976, 0.992, 0.960, 0.990, 0.992, 0.901, 0.815, 0.947, and 0.504, indicating that the similarities of 19 samples showed a similar pattern with the exception of sample 20. Sample S20 could be considered adulterated. This was further confirmed by principal component analysis and hierarchical clustering analysis. The HPLC fingerprints of different Chaenomelis Fructus had obvious differences in area of common peaks, but less differences in the number of common peaks.

**Conclusions:**

The chromatographic fingerprint of Chaenomelis Fructus with high characteristics and specificity can be used as a reference to control its quality, providing a fast quality evaluation tool for distinguishing between the authentic Chaenomelis Fructus and the adulterated products.

## Introduction

Chaenomelis Fructus is widely cultivated in China, and their fruits are always harvested at the beginning of their ripening stage. Chaenomelis Fructus is mainly produced in Sichuan, Anhui, Hubei, Chongqing, Shandong and other places, and because of the climate, the harvest time of those areas is different. The harvest time of Xuan mugua is the first 10 days of July, and the middle 10 days of July and August are the harvest time of Ziqiu mugua and Chuan mugua, respectively. More and more Chaenomelis are planted for the food industry to produce fruit juice, fruit vinegar, fruit tea, canned fruit and candied fruit. Simultaneously, from the viewpoint of medicine food homology, Chaenomelis Fructus is also a famous type of traditional Chinese medicine. In the present investigation, many researchers have confirmed that it has good antioxidant, antihyperlipidemic [1], antitumor [2], anti-inflammatory, anti-influenza viral [3, 4] and anti-Parkinson [5] activities.

Triterpenes (oleanolic acid and ursolic acid), flavonoids (quercetin, luteolin, catechin, epicatechin, procyanidin, B1, and B2), phenolics (chlorogenic acid), carbohydrates, amino acids, proteins, gallic acid, protocatechuic acid, caffeic acid, syringic acid, and tannins are active compounds in Chaenomelis [6–10], particularly oleanolic acid and ursolic acid are two active compounds legally designated in Pharmacopoeia of the People’s Republic of China [11], and their contents are used for the quality control of Chaenomelis herbal medicine.

As is known to all, the Chaenomelis implanted in Xuancheng City, Anhui Province, Changyang County, Hubei Province, Qijiang County, and Chongqing City was investigated. The Chaenomelis of Anhui Xuancheng is the top grade. However, Chaenomeles in the Chinese medicine market often have a variety of sources, causing *Chaenomeles sinensis*  (Thouin) Koehne quality problems such as light Chaenomelis replacement. Therefore, it is difficult to tell whether the processed Chaenomelis on the market is authentic or adulterated, which adds difficulty to the identification of Chinese medicinal materials. Thus, it is essential to develop a type of quality assessment system that comprehensively analyzes the complex components of Traditional Chinese Medicine (TCM). Chromatographic fingerprint is an effective identification method for the quality control of TCM [12–14].

## Experimental

### Materials and reagents

Twenty batches of Chaenomelis Fructus samples were collected from different regions of China for analysis, and the source information is listed in the supplemental Table 1. The authentication of the samples was identified by Dr. Dequn Wang according to the morphological features, and the voucher specimens were deposited in the Anhui University of Chinese Medicine.

**Table 1:** Sample source table

Sample	Source	Place of purchase
S1	Changyang Hubei	Anhui Xiehe City Pieces Co., Ltd.
S2	Changyang hubei	Anhui Xiehe City Pieces Co., Ltd.
S3	Hubei	Bozhou Huqiao Pharmaceutical Industry
S4	Hubei	Bozhou Huqiao Pharmaceutical Industry
S5	Xuancheng	Wansheng Chinese Medicine Pieces Co., Ltd.
S6	Hubei	Yonggang Pieces Factory Co., Ltd.
S7	Guangxi	Beijing Tong Ren Tang pieces co., Ltd.
S8	Guangxi	Beijing Tong Ren Tang pieces co., Ltd.
S9	Sichuan	Anhui Xintai Pharmaceutical Co., Ltd.
S10	Hubei	Traditional Chinese Medicine Pieces Factory of Jingwan
S11	Hubei	Traditional Chinese Medicine Pieces Factory of Jingwan
S12	Sichuan	Chinese Herbal Pieces Co., Ltd. of Anhui Huashantang
S13	Xuancheng	Chinese Herbal Pieces Co., Ltd. of Anhui Zhiliang
S14	Hubei	Chinese Herbal Pieces Technology Co., Ltd
S15	Hubei	Puren Chinese Medicine Pieces Co., Ltd.
S16	Hubei	Bozhou Kangmei Medicinal Materials Market
S17	Xuancheng	Bozhou Kangmei Medicinal Materials Market
S18	Sichuan	Bozhou Kangmei Medicinal Materials Market
S19	Sichuan	Bozhou Kangmei Medicinal Materials Market
S20	Yunnan	Bozhou Kangmei Medicinal Materials Market

**Fig. 1:**
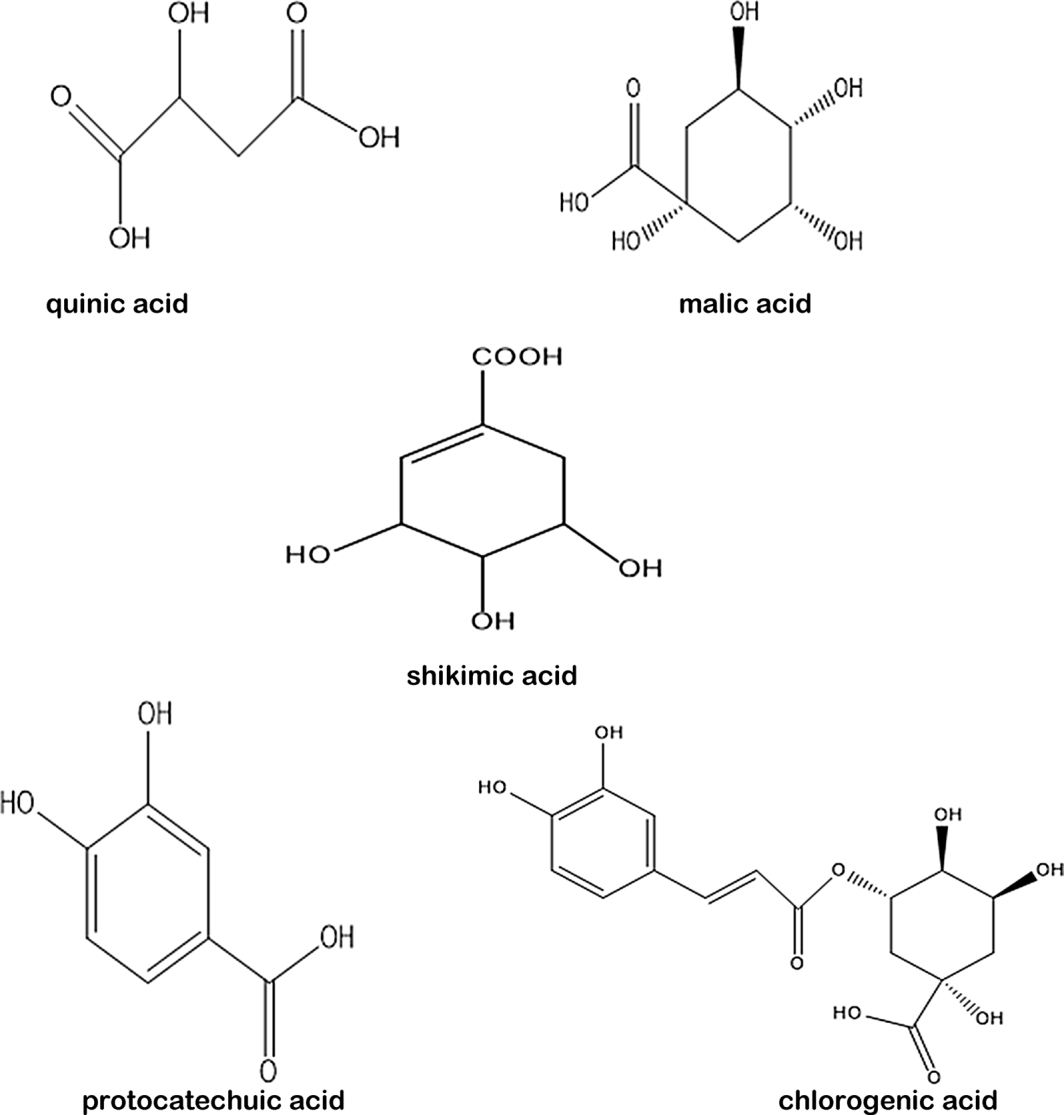
Chemical structures of the reference compounds

Reference compounds of quinic acid, malic acid, protocatechuic acid, shikimic acid and chlorogenic acid were provided by Cloma Biotechnology Corporation (Chengdu), and all five reference compounds used in the analysis had purities ≥ 98%. Their chemical structures are shown in Fig. 1.

Acetonitrile of chromatographic grade was purchased from Runjie-Corporation (Ocepak, Germany) and deionized water obtained from a Milli-Q water purification system (Millipore, Bedford, MA, USA) was used for the preparation of the mobile phase. The other organic reagents were of analytical grade.

### Apparatus

High-performance liquid chromatography (Dai’an U3000); JA2103N electronic balance (1/1000, German Sartorius company); BP211D electronic balance (1/100,000, German Sartorius); LKTC-L type electrothermal constant temperature water bath (Changzhou Tian Rui Instrument Co., Ltd.); KQ-250DB type numerical control ultrasonic cleaner (Kunshan City Ultrasonic Instrument Co, Ltd.); HS-3CA precision acidity meter (Shanghai Da Pu Instrument Co, Ltd.); QE-200 type high speed crusher (Zhejiang erect industry and Trade Co., Ltd.); RE-52AA rotary evaporator (Shanghai Yarong Biochemical Instrument Factory).

### HPLC–DAD instrumentation and conditions

The HPLC analysis was carried out by a Dionex UltiMate 3000 LC series–diode array detector (DAD) system, which was used for acquiring chromatograms and ultraviolet (UV) spectra. An UltimateAQ analytical column (4.6 mm × 250 mm, 5 µm) was used for the HPLC analysis.

The detection wavelength used was as follows: 210 nm from 0 to 21 min, 260 nm from 21 to 40 min, 325 nm from 40 to 60 min, and the column temperature was set at 30 °C. The mobile phase consisted of acetonitrile (B) and water containing 0.04% (v:v) phosphoric acid (A). A gradient program was optimized as follows: 0 min, 100:0 (A:B, v/v); 10 min 100:0 (A:B, v/v); 25 min 94:6 (A:B, v/v); 35 min 94:6 (A:B, v/v); 40 min 85:15 (A:B, v/v); 60 min 85:15 (A:B, v/v). The flow rate was 0.6 mL/min. The injection volume of the samples and the standard solutions were both 10 µL.

### Preparation of the standard solution

Five compounds were separately weighted and dissolved in water containing 0.04% (v:v) phosphoric acid (A) as the stock standard solution. Then, appropriate volumes of each stock solution were mixed together to produce a solution containing 1.435 mg/mL quinic acid, 1.547 mg/mL malic acid, 0.211 mg/mL protocatechuic acid, 0.290 mg/mL shikimic acid, and 0.204 mg/mL chlorogenic acid, which was used as the reference solution.

### Preparation of the sample solution

Chaenomelis Fructus samples were vacuum-dried in an oven at 50 °C and were ground into fine powder (50 mesh) using a powdering machine. Subsequently, Chaenomelis Fructus samples powder (2 g) was accurately weighed and placed into a 25 mL Erlenmeyer flask. After 40 mL of 50% ethanol was added and stood for 10 min, the mixture was extracted for 60 min by ultrasound (100 W). The operation was repeated once, and the residue was washed with 20 mL of water and then acidified to PH 2.00 with 1 mol/L hydrochloric acid. Then, the residue was extracted twice with *n*-butanol of an equal volume. The total extracts were recovered by solvent decompression, which were then filled up to the calibration mark with the mobile phase (0.04% phosphoric acid solution). The extracts were then filtered through a microfiltration membrane (0.45 μm) to obtain the sample solution.

### Validation of the HPLC method

The Chaenomelis Fructus sample (sample 13) was used in the validation test. The precision was determined by injecting the same sample solution six times. The repeatability was determined by analyzing six independent sample solutions extracted from the same batch of Chaenomelis Fructus. The stability test was evaluated by injecting the same sample solution at 0, 2, 4, 6, 8, 10, 12 and 24 h after preparation. The Chaenomelis Fructus samples were analyzed, and the chromatograms were recorded.

### Data analysis

The data analysis was processed by the professional software Similarity Evaluation System for Chromatographic Fingerprint of Traditional Chinese Medicine (Version 2004A), which was recommended by the State Food and Drug Administration (SFDA) of China [15]. This software was used to calculate the correlation coefficients of the chromatographic profiles of 20 batches of Chaenomelis Fructus samples and generate the simulative mean chromatogram. The similarities of different chromatographic fingerprints were compared with the simulative mean chromatogram. These data were standardized before HCA and PCA. The first and second principal components were selected to draw a scatter plot for PCA. The average linkage between groups method was applied for HCA, with the square Euclidean distance used to measure the distance matrix between observations [16]. The HCA and PCA of all the samples were performed using the SPSS software (IBM SPSS Statistics 19, Armonk, New York, USA).

## Results and discussion

### Optimization of the mobile phase

Different mobile phase compositions such as methanol–phosphoric acid aqueous solution, methanol–water and acetonitrile–water systems were compared. The results demonstrated that the 0.4% acetonitrile–phosphoric acid aqueous solution exhibited a satisfactory resolution, moderate retention time and smooth baseline, which was adopted as the mobile phase in this study.

### Optimization of the column temperature

The effect of the column temperature (25, 30 and 35 °C) on the chromatographic peak separation was investigated, and it was found that the resolution of the peaks became better at 30 °C by HPLC. Thus, 30 °C was used by HPLC.

### Identification of the common peaks

**Fig. 2:**
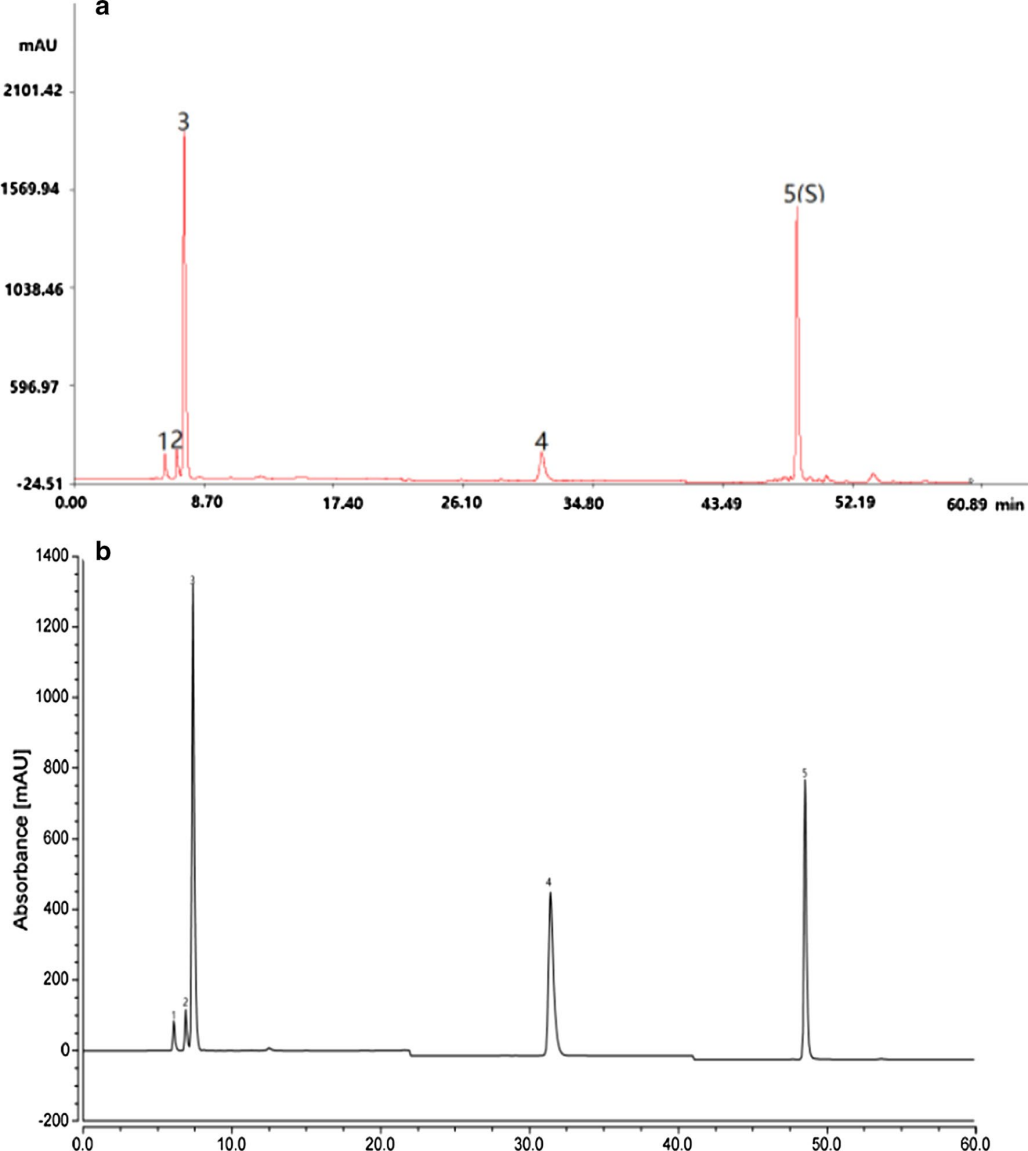
**a** The reference fingerprint of Chaenomelis Fructus showing 5 common peaks. **b** HPLC chromatographic profile of four reference substances Peak 1 quinic acid; Peak 2 malic acid; Peak 3 shikimic acid; Peak 4 protocatechuic acid; Peak 5 chlorogenic acid

The HPLC fingerprints generated by the 20 batches of Chaenomelis Fructus samples were analyzed and 5 common peaks were found Fig. 2a). Among them, five common peaks were identified using the developed HPLC method based on the comparison of their retention time with the reference substances. Peak 1, 2, 3, 4, and 5 were identified as quinic acid, malic acid, protocatechuic acid, shikimic acid and chlorogenic acid, respectively Fig. 2b).

### Validation of the HPLC fingerprint method

#### Precision test

For the precision study, the retention time and the peak area of peak 5 (chlorogenic acid) were chosen as the reference, and the relative retention time (RRT) and the relative peak area (RPA) of the five common peaks of all the samples were calculated. The relative standard deviation (RSD) of the RRT of each common peak was found to be less than 3% Table 2, which showed that the precision of the HPLC fingerprint method was good.

**Table 2:** The precision, repeatability and stability of the common peaks in Chaenomelis Fructus

Peak no.	Precision (RSD, %)		Repeatability (RSD, %)		Stability (RSD, %)	
	RRT	RPA	RRT	RPA	RRT	RPA
1	0.32	1.07	0	0.70	0.37	1.10
2	0.29	1.02	0	0.97	0.32	0.89
3	0.27	0.33	0	0.10	0.23	0.53
4	0.17	0.40	0	0	0.16	0.46
5 (S)	–	–	–	–	–	–

#### Repeatability test

The RRT and RPA of the five common peaks were calculated in the repeatability test. The RSD of the RRT for each peak was less than 0.14%. The two RSD prompted that the repeatability of the HPLC method was satisfactory.

#### Stability test

For the stability test, the sample solution was measured at 0, 2, 4, 6, 8, 10, 12 and 24 h after preparation, and then the RRT and the RPA were calculated. The RSD of the RRT was found to be less than 3%. The results showed that the Chaenomelis Fructus sample solution was stable within 24 h.

#### Similarity analysis

20 Chaenomelis Fructus samples acquired from various sources were analyzed with the established methods and the HPLC fingerprint profiles were obtained. Five coexisting fingerprint peaks were found in all the analyzed samples.

The RRT and RPA of these 5 peaks were calculated using the “Similarity Evaluation System” for Chromatographic Fingerprint of Traditional Chinese Medicine (Version 2004A, Chinese Pharmacopoeia Commission, Beijing, China), and the results are summarized in Tables 3 and 4, respectively. The RSD of the RRT was found to be less than 3%, demonstrating that the fingerprint analysis was more precise. The RSD of the RPA were relatively larger. These results indicated that the retention time of the common peaks was consistent among batches, but the contents of the components among batches varied.

**Table 3:** RRT of common peaks in the 20 batches of Chaenomelis Fructus samples

Peak no.	RRT				
	1	2	3	4	5
1	0.126	0.142	0.152	0.641	1
2	0.125	0.141	0.151	0.646	1
3	0.126	0.142	0.152	0.646	1
4	0.126	0.142	0.152	0.646	1
5	0.125	0.142	0.152	0.646	1
6	0.126	0.142	0.153	0.646	1
7	0.125	0.142	0.152	0.645	1
8	0.125	0.142	0152	0.646	1
9	0.126	0.142	0.152	0.647	1
10	0.126	0.142	0.153	0.648	1
11	0.126	0.142	0.152	0.647	1
12	0.125	0.142	0.152	0.646	1
13	0.125	0.142	0.152	0.647	1
14	0.126	0.142	0.152	0.647	1
15	0.126	0.142	0.152	0.649	1
16	0.126	0.142	0.152	0.649	1
17	0.125	0.141	0.152	0.645	1
18	0.125	0.142	0.152	0.644	1
19	0.125	0.141	0.152	0.646	1
20	0.125	0.142	0.152	0.647	1
Mean	0.126	0.142	0.152	0.646	1
RSD (%)	0.41	0.26	0.26	0.27	0

**Table 4:** RPA of common peaks in the 20 batches of Chaenomelis Fructus samples

Peak no	RPA				
	1	2	3	4	5
1	0.075	0.057	1.178	0.156	1
2	0.070	0.119	1.316	0.142	1
3	0.119	0.095	1.526	0.126	1
4	0.073	0.072	1.080	0.177	1
5	0.081	0.073	0.877	0.208	1
6	0.057	0.05	0.889	0.127	1
7	0.080	0.101	1.318	0.098	1
8	0.133	0.468	1.068	1.021	1
9	0.098	0.059	0.152	0.228	1
10	0.120	0.385	0.889	1.043	1
11	0.059	0.063	0.949	0.110	1
12	0.056	0.049	0.662	0.152	1
13	0.09	0.074	1.115	0.238	1
14	0.055	0.044	0.564	0.271	1
15	0.015	0.017	0.213	0.032	1
16	0.080	0.054	0.896	0.338	1
17	0.126	0.475	1.051	1.063	1
18	0.154	0.828	1.204	1.158	1
19	0.091	0.432	0.676	0.447	1
20	0.133	0.164	0.056	2.148	1
Mean	0.088	0.184	0.884	0.464	1
RSD (%)	38.63	117.35	44.96	116.96	0

*RRT* relative retention time, *RPA* relative peak area

**Fig. 3:**
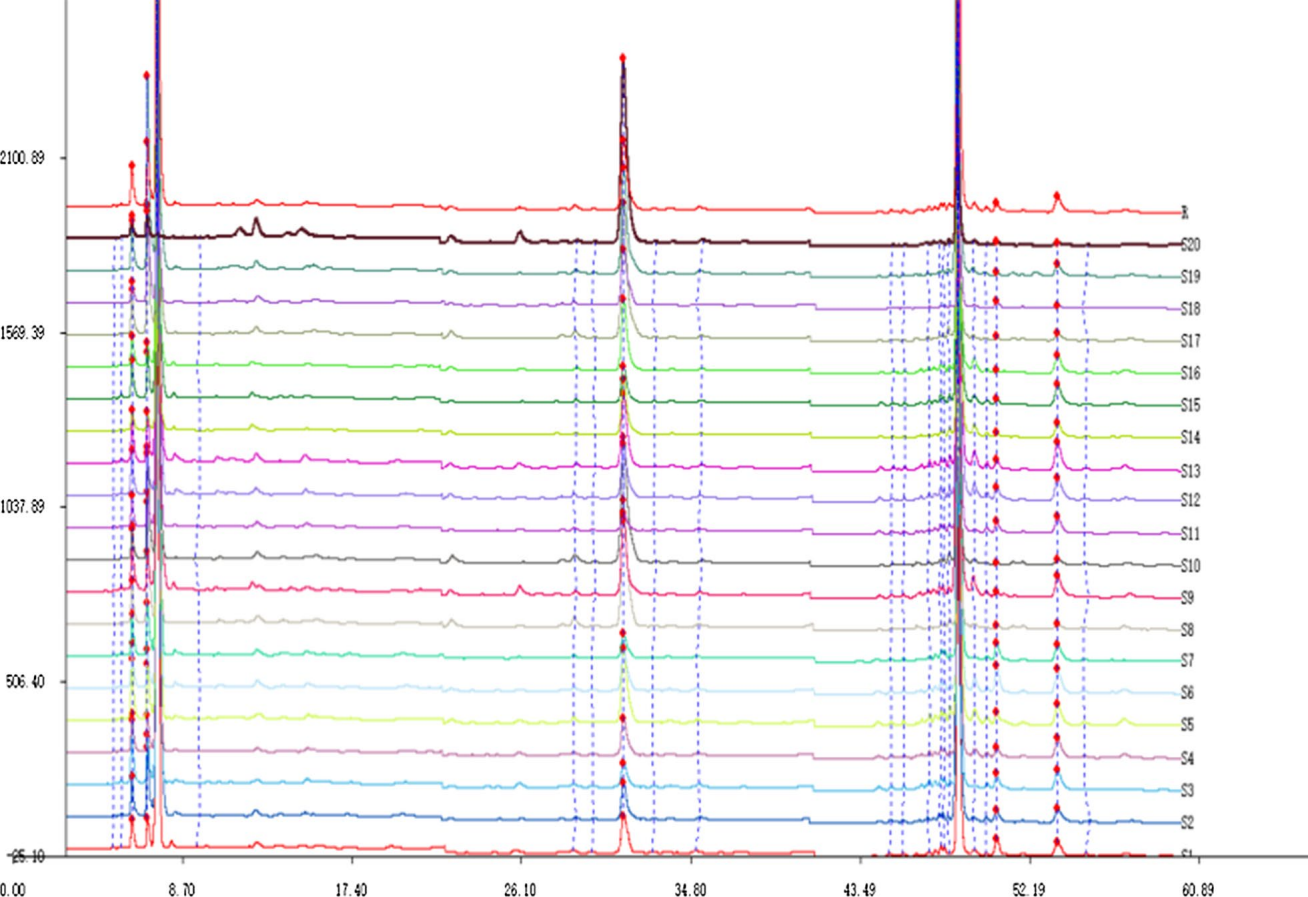
HPLC fingerprints of the 20 batches of Chaenomelis Fructus. S1–S20 represent samples numbered from 1 to 20

The chromatographic fingerprints of the 20 Chaenomelis Fructus samples are shown in Fig. 3, and the results of the similarity analysis are listed in Table 5. The similarity results of the 20 samples are in the range of 0.504–0.994, and S20 displayed a relatively low similarity, with a similarity value of 0.504. This difference proved that the source of S20 is not *Chaenomeles speciosa* (Sweet) Nakai.

**Table 5:** Similarities of the 20 batches of Chaenomelis Fructus

Sample no.	Similarity
S1	0.967
S2	0.979
S3	0.965
S4	0.992
S5	0.994
S6	0.988
S7	0.974
S8	0.909
S9	0.993
S10	0.894
S11	0.983
S12	0.976
S13	0.992
S14	0.960
S15	0.990
S16	0.992
S17	0.901
S18	0.815
S19	0.947
S20	0.504

The results of the similarity analysis showed that the chemical types of the Chaenomelis Fructus samples from different regions, except for Yunnan, were basically the same; however, the relative contents of each component may vary in some of the samples. This finding demonstrated that the present HPLC fingerprint method could distinguish the adulterants of Chaenomelis Fructus.

### Hierarchical cluster analysis (HCA) and principal component analysis (PCA)

The hierarchical cluster analysis and the principal component analysis in this study were performed based on the relative peak areas of those common characteristic peaks calculated by the Similarity Evaluation System.

**Fig. 4:**
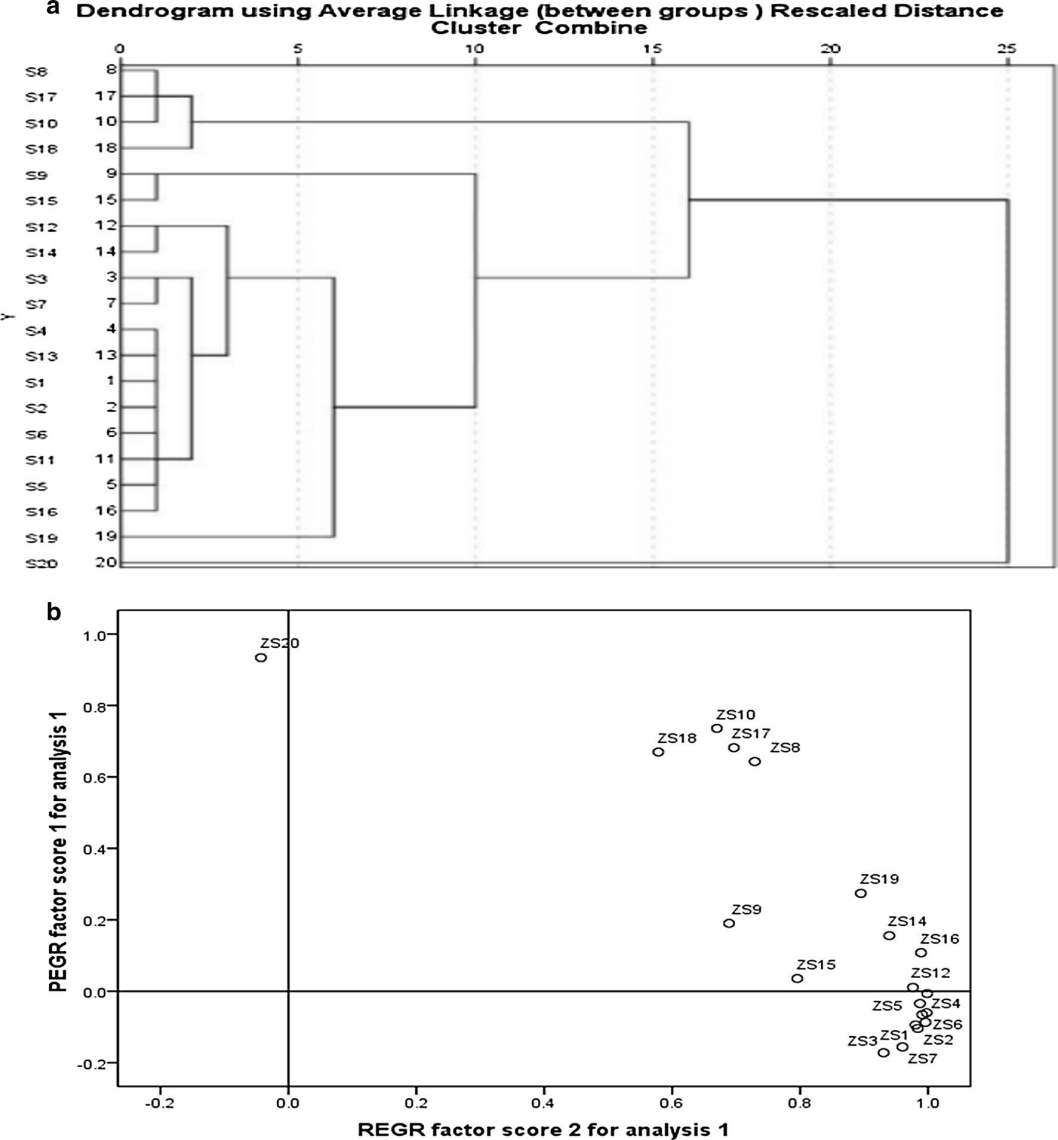
Results of the hierarchical cluster analysis and the principal component analysis of the samples from 20 provinces. **a** Hierarchical cluster analysis; **b** principal component analysis

To assess the similarity and the differences between various samples, Q-cluster analysis was applied to sort the 20 Chaenomelis Fructus samples into two groups. A dendrogram of HCA is shown in Fig. 4a, which shows that the 20 samples are classified into two quality clusters. Among them, sample 20 is in Cluster A and the others are in Cluster B, indicating the significant difference between Chaenomelis Fructus from Yunnan and other varieties. To evaluate the homogeneity of the quality of the Chaenomelis Fructus samples from different regions of China, PCA was performed. The scatter points in Fig. 4b showed that the results were consistent with those of HCA, and Yunnan samples could be distinguished through the scatter diagram.

## Conclusion

A HPLC method for the profiling of Chaenomelis Fructus has been established and validated. Twenty batches of raw material samples of Chaenomelis Fructus from various cultivation locations were evaluated and 5 coexisting peaks were found in the 20 samples. The 20 batches of these samples were analyzed simultaneously, and the developed method exhibited good stability, precision and repeatability. Based on the present study, this method lays a solid foundation for the quality control of Chaenomelis Fructus and will serve as a valuable reference for the quality evaluation and standardization of Chinese herbal medicine.

## Abbreviations

HPLC: high-performance liquid chromatography
TCM: Traditional Chinese Medicine
RRT: relative retention time
RPA: relative peak area
RSD: relative standard deviation
DAD: diode array detector
UV: ultraviolet
SFDA: State Food and Drug Administration
SMC: simulative mean chromatogram
